# Eye movements reveal sexually dimorphic deficits in children with fetal alcohol spectrum disorder

**DOI:** 10.3389/fnins.2015.00076

**Published:** 2015-03-11

**Authors:** Angelina Paolozza, Rebecca Munn, Douglas P. Munoz, James N. Reynolds

**Affiliations:** ^1^Centre for Neuroscience Studies, Queen's UniversityKingston, ON, Canada; ^2^Department of Biomedical and Molecular Sciences, Queen's UniversityKingston, ON, Canada

**Keywords:** fetal alcohol spectrum disorder, eye movements, saccades, biomarkers, sexual dimorphism, saccade dysmetria

## Abstract

**Background:** We examined the accuracy and characteristics of saccadic eye movements in children with fetal alcohol spectrum disorder (FASD) compared with typically developing control children. Previous studies have found that children with FASD produce saccades that are quantifiably different from controls. Additionally, animal studies have found sex-based differences for behavioral effects after prenatal alcohol exposure. Therefore, we hypothesized that eye movement measures will show sexually dimorphic results.

**Methods:** Children (aged 5–18 years) with FASD (*n* = 71) and typically developing controls (*n* = 113) performed a visually-guided saccade task. Saccade metrics and behavior were analyzed for sex and group differences.

**Results:** Female control participants had greater amplitude saccades than control males or females with FASD. Accuracy was significantly poorer in the FASD group, especially in males, which introduced significantly greater variability in the data. Therefore, we conducted additional analyses including only those trials in which the first saccade successfully reached the target within a ± 1° window. In this restricted amplitude dataset, the females with FASD made saccades with significantly lower velocity and longer duration, whereas the males with FASD did not differ from the control group. Additionally, the mean and peak deceleration were selectively decreased in the females with FASD.

**Conclusions:** These data support the hypothesis that children with FASD exhibit specific deficits in eye movement control and sensory-motor integration associated with cerebellar and/or brain stem circuits. Moreover, prenatal alcohol exposure may have a sexually dimorphic impact on eye movement metrics, with males and females exhibiting differential patterns of deficit.

## Introduction

Prenatal alcohol exposure can cause damage to the developing brain of the fetus and may lead to a range of cognitive deficits that include problems with executive functions, attention, and working memory (Mattson et al., 1999; Rasmussen, 2005; Kodituwakku, 2009). This can lead to negative behavioral, neuropsychiatric, and maladaptive outcomes commonly observed in this population, which has recently gained greater attention, as neurodevelopmental disorder associated with prenatal alcohol exposure was added to the fifth edition of the Diagnostic and Statistical Manual of Mental Disorders (Streissguth et al., 2004; Rasmussen et al., 2008; American Psychiatric Association, 2013). Fetal alcohol spectrum disorder (FASD) is an umbrella term used to describe the full range of adverse effects induced by prenatal alcohol exposure. FASD has several subtypes that include fetal alcohol syndrome (FAS) and partial fetal alcohol syndrome (pFAS), which presents with full/partial facial dysmorphology, growth deficiency, and central nervous system dysfunction; and alcohol related neurodevelopmental disorder (ARND) which presents with central nervous system dysfunction (Chudley et al., 2005). Many secondary disabilities have been identified in those diagnosed with FASD including mental health disorders, addictions, trouble with the law, and problems with employment (Pei et al., 2011). Two key protective factors that have been identified as decreasing secondary disabilities include receiving services for developmental disabilities and having a diagnosis before the age of 6 years (Streissguth et al., 2004). Effective screening tools that can accurately assess brain function in a non-invasive manner could facilitate the early and accurate identification of alcohol-exposed children. Also, by identifying the underlying brain pathology better interventions and services can be developed. Here, we assess eye movement measures obtained during the performance of a visually-guided saccade task as a possible screening tool for use in children with FASD.

Studies conducted in animal models have found behavioral and physiological sex differences in offspring as a consequence of prenatal alcohol exposure. These studies have found differences in male and female physiological responses to stressors, serotonin, hypothalamic-pituitary-adrenal (HPA) axis function, and behavioral responses in recognition memory and spatial working memory in animals prenatally exposed to alcohol (Weinberg and Jerrells, 1991; Goodlett and Peterson, 1995; Weinberg et al., 2008; Kelly et al., 2009; Sliwowska et al., 2014). For example, female rats prenatally exposed to alcohol show deficits in their ability to use or respond to environmental cues (Weinberg, 1988, 1992b), which could translate into deficits in visual response processing. Additionally, several studies have found differences in exploratory eye movements between control males and females when viewing natural images (Nishiura et al., 2007; Mercer Moss et al., 2012). Based on these findings, we sought to investigate if any behavioral eye movement measures displayed an interaction between males and females in control and FASD participants. Due to the above differences found in animal studies and the high precision eye movement control measures have when assessing brain damage, we predicted that the eye movement measures of healthy controls and those with FASD will show sexually dimorphic results which can be used to better characterize children prenatally exposed to alcohol.

Saccades are rapid eye movements that bring visual targets onto the fovea of the retina. This tool was chosen to assess children with FASD because the measurement of eye movements can be used to differentiate disorders of the nervous system by assessing sensory, motor, and cognitive function (Munoz et al., 2007; Ramat et al., 2007). The eye movement system has also been used to characterize healthy development throughout childhood (Munoz et al., 1998; Luna et al., 2001, 2008; Alahyane et al., 2014). Important features of saccadic eye movements are the metrics, which describe the accuracy and quality of the motor processes. One of the most commonly studied features of saccade metrics is the main sequence which examines the relationship between amplitude, velocity and duration (Bahill et al., 1975; Leigh and Zee, 2006). Examining metric measures and main sequence relationships may reveal important information about brain function, as the brain regions involved are well-characterized (Garbutt et al., 2001; Scudder et al., 2002; Sparks, 2002; Leigh and Zee, 2006).

Eye movement control is a reliable and accurate measure of prenatal alcohol exposure and can differentiate those with FASD from typically developing controls (Green et al., 2007, 2009; Paolozza et al., 2013, 2014a,b; Tseng et al., 2013a,b). One task that has revealed differences between FASD and control participants is the prosaccade task which requires participants to make visually-guided saccades to peripheral targets (Munoz and Everling, 2004; Johnson et al., 2012). In our previous studies, children with FASD were shown to have significantly poorer accuracy in the prosaccade task, with more variable saccade endpoints, and increases in saccade endpoint deviation and the frequency of additional, corrective saccades required to achieve final fixation (Paolozza et al., 2013). Moreover, this increased saccade endpoint deviation correlated with poorer visuospatial processing on a psychometric task of line orientation judgment (Paolozza et al., 2014b). Therefore, reduced ability to control saccade accuracy is an important feature of the FASD behavioral phenotype. Here, we investigate the metrics of visually-guided saccades in a large group of both children with FASD and healthy controls to test the hypothesis that children with FASD will exhibit dysfunction in the cerebellum and/or brainstem components of the saccade control circuit, as evidenced by deviations from normal saccade metrics. This study had two objectives: to examine group differences in saccade metrics in children with FASD compared to healthy controls and to test for sex differences in these two groups.

## Materials and methods

### Participants

Participants aged 5–18 years were recruited from five sites across Canada. Children with FASD (*n* = 71; mean age 11.8 ± 0.4) were previously assessed and diagnosed according to the Canadian Guidelines for FASD Diagnosis (Chudley et al., 2005) and were recruited through diagnostic clinics in Kingston, ON; Ottawa, ON; Edmonton, AB; Cold Lake, AB; and Winnipeg, MB, as part of a larger Canada-wide network study funded by NeuroDevNet (Reynolds et al., 2011). Typically developing children (*n* = 113; mean age 10.3 ± 0.3) were recruited from the same geographical areas and were excluded if they had any neurological or psychiatric disorder, or visual disturbance, other than requiring corrective lenses. All experimental procedures were reviewed and approved by the Human Research Ethics Boards at Queen's University (Kingston), University of Alberta (Edmonton and Cold Lake), Children's Hospital of Eastern Ontario (Ottawa), and the University of Manitoba (Winnipeg). Written informed consent was obtained from a parent or legal guardian and assent was obtained from each child before study participation. Demographic information is summarized in Table 1. Socioeconomic status (SES) was calculated using Hollingshead's Four-Factor Index of Social Status (Hollingshead, 2011). Study data were collected and managed using REDCap electronic data capture tools (Harris et al., 2009).

**Table 1:** **Demographic variables for control and FASD groups**.

	**Control (*n* = 113)**	**FASD (*n* = 71)**	***p*-value**
**SEX**			
Males (%)	53 (47)	39 (55)	0.65
Females (%)	60 (53)	32 (45)	0.38
**SUBTYPE**			
Fetal alcohol syndrome (%)	–	8 (11)	
Partial fetal alcohol syndrome (%)	–	14 (20)	
Alcohol related neurodevelopmental disorder (%)	–	49 (69)	
**COMORBIDITIES**			
Attention deficit hyperactivity disorder (%)	–	43 (61)	
Anxiety (%)	–	9 (13)	
Oppositional defiant disorder (%)	–	7 (10)	
Depression (%)	–	6 (8)	
Other (%)	–	19 (26)	
**MEDICATIONS**			
Stimulants (%)	–	31 (43)	
Antipsychotics (%)	–	17 (24)	
Antidepressants (%)	–	8 (11)	
Other (%)	–	14 (19)	
**OTHER**			
Age (years ± SD)	10.4 ± 0.3	11.7 ± 0.4	0.0009
Socioeconomic status	47 ± 7	41 ± 14	0.0096
**ETHNICITY**			
First Nations/Metis (%)	2 (2)	43 (61)	<0.0001
Caucasian (%)	106 (94)	25 (35)	<0.0001
Other (%)	5 (4)	3 (4)	Ns

*The control group had no comorbidities or medications except asthma and requiring an inhaler (n = 3)*.

### Saccadic eye movement recordings

Participants were seated comfortably in a dark, quiet room on a stationary chair and instructions for each trial were given verbally, and repeated back to the experimenter by the participant. Eye position was recorded using the Eyelink 1000 (SR Research, Kanata, Canada). A 17” LCD monitor and mounted infrared camera were at a distance of 58–64 cm from the left eye. The position of the left pupil was digitized in both the vertical and horizontal axes at a sampling rate of 500 Hz. Saccades were defined as having a speed of greater than 2.5 times the standard deviation of the background noise (measured during fixation) for at least 5 sample points. Before each task the eye movements of each participant were calibrated using nine screen targets (eight around the periphery and one central) of known position. This ensured that the participants had no visual disturbances that would impair task performance as they would be unable to orient their eyes to the target positions correctly.

Each trial started with illumination of a central fixation point (FP) for 800–1200 ms. The FP then disappeared and, after a 200 ms delay (gap period), a peripheral target appeared randomly at 10° to the left or right of the FP. Participants were given 1000 ms to initiate and complete a saccade toward the target. No feedback was given about performance. The gap period was employed because it produces the shortest saccadic reaction time (SRT) (Saslow, 1967; Fischer and Ramsperger, 1984; Dorris and Munoz, 1995). One block of 60 trials was obtained from each participant as part of a larger battery of eye movement and psychometric tests. The entire testing session was 2 h and participants were compensated with gift cards. Testing was kept to a maximum of 2 h to minimize fatigue. We have previously reported on other measures of this battery in this cohort (Paolozza et al., 2013, 2014a,b).

### Data analysis

Data were analyzed using custom software developed in MATLAB (Mathworks, Natick, Massachusetts). Only correct trials in which the participants fixated on the FP at the start of the trial and made a saccade in response to the target appearance were included in the analysis. On average 91% of trials were viable (53–100% range).

For correct trials, SRT was defined as the time from the appearance of the peripheral target to the initiation of the first saccade. The deviation of the saccade endpoint was defined as the angular distance between the ideal path from fixation to target and the trajectory of the first saccade toward the goal by drawing a straight line from the beginning to the end of the saccade (Paolozza et al., 2013). Amplitude, peak velocity, duration, peak acceleration, and peak deceleration were calculated for each correct trial. The mean acceleration (the slope of the velocity plot from saccade onset to peak velocity) and mean deceleration (the slope from peak velocity to saccade termination) were also calculated. A skew index was calculated from mean acceleration (slope 1) and deceleration (slope 2) using the following equation: skew index = (slope 1 − slope 2)/(slope 1 + slope 2). A positive skew indicated that mean acceleration (slope 1) was steeper than mean deceleration (slope 2), whereas a negative skew indicated that mean deceleration (slope 2) was steeper than mean acceleration (slope 1). A skew index of 0 indicated that both slopes were the same.

A separate analysis was performed to explore potential covariates associated with demographic factors (Table 1) by first examining the data for trends/disparities and then running the appropriate statistical tests. All data were also examined for outliers and if a data point was found to be greater than two standard deviations away from the mean of that group they were excluded.

The control group data were first analyzed by performing Pearson correlations between each saccade measure and age. If the measure varied significantly with age, then age corrections were performed. Due to the large number of control participants it was possible to perform age-correction by calculating a standardized *t*-score equation for each individual age. Age-corrected scores for the FASD group were then calculated using the *t*-score equation obtained from the control group. Interactions between sex and group were analyzed using Two-Way, two-tailed repeated measures analysis of variance (ANOVA). Tukey's *post-hoc* test for multiple comparisons with adjusted *p*-values was used for those outcomes in which a group difference and interaction were found to compare all groups with each other. Sidak's *post-hoc* test for multiple comparisons with adjusted *p*-values was used for only those outcomes in which an interaction was found to compare the control and FASD group. Pearson correlations between the main sequence measures were also performed using individual trial data to allow for a range of amplitudes to be measured. The correlation coefficients were then compared between the FASD and control group by using the Fisher r-to-z transformation.

In the current dataset, the accuracy of the saccade endpoint and amplitude were significantly poorer in the FASD group, which introduced significantly greater variability and statistically greater variances. Therefore, we conducted additional analyses including only those saccades in which amplitude and endpoint fell within 9 ± 1° on the horizontal axis and ±1° on the vertical axis. These values were selected because they reflected the mean values of control participants, fitting with the tendency for initial saccades to be slightly hypometric (Leigh and Zee, 2006). Interactions between sex and group were analyzed as described above for the unrestricted data using Two-Way ANOVA.

## Results

### Demographic findings

Differences in sex distribution between the two groups were calculated by performing Fisher's exact test for both groups and no significant differences were found. FASD subtype was investigated by dividing the FASD group into two subgroups (FAS/pFAS and ARND) and comparing these subgroups to the control group on all outcome measures using a One-Way ANOVA. No significant differences were detected indicating the ARND group performed similarly to the FAS/pFAS group. Due to the relatively low number of most comorbidities in the FASD group, only attention deficit hyperactivity disorder (ADHD) could be properly investigated. This was accomplished by dividing the FASD group into those with ADHD and those without and comparing the two groups on the metric measures using a *t*-test. No significant differences were found indicating that a comorbidity of ADHD did not affect the data for this cohort. Age was analyzed via a *t*-test and the control group was found to be significantly younger than the FASD group [*t*_(179)_ = 3.37, *p* = 0.0009]. However, we controlled for age by performing age corrections on those outcome measures that changed significantly with age. Next, SES was investigated using a *t*-test to compare the two groups. The FASD group was found to have significantly lower SES compared to controls [*t*_(125)_ = 2.63, *p* = 0.0096]. However, when correlations were run between SES score and each eye movement score no significant relationships were detected indicating that SES did not affect metric scores in this cohort. Finally, ethnicity was examined in the same way as ADHD by dividing the FASD group into those identified as First Nations and those with any other ethnicity (primarily Caucasian). Using a *t*-test, no significant differences were found between the two groups. Therefore, diagnostic subgroup, comorbidities, SES, and ethnicity did not influence the data for this cohort and did not need to be included as covariates.

### Overall metric findings

**Figure 1:**
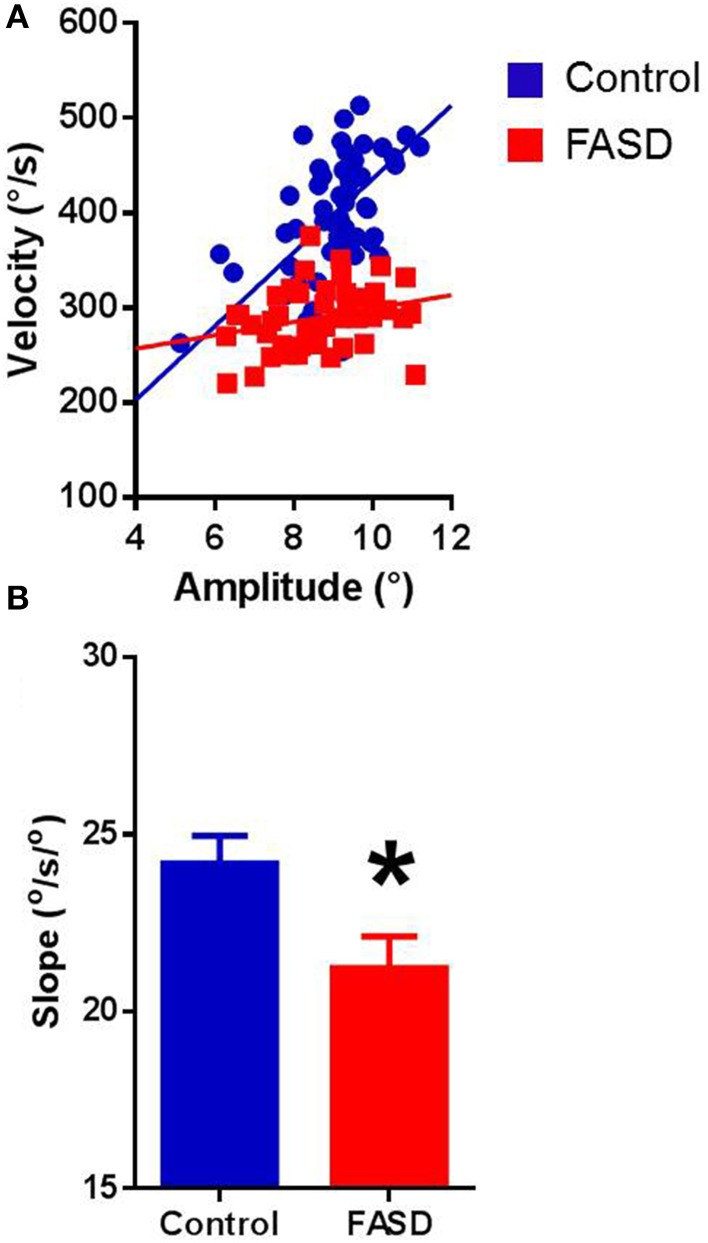
**Main sequence relationships. (A)** The velocity-amplitude relationship of a 15-year-old control participant and 15-year-old FASD participant. **(B)** Data for participants in the FASD group (*n* = 71) shown in red and the control group (*n* = 113) shown in blue. The slope of the velocity-amplitude relationship was significantly lower in the FASD group. Control is shown in blue and FASD is shown in red. ^*^p < 0.05.

In both typically-developing and FASD participants, the best-fit lines of the main sequence relationships were linear for each participant (Figure 1A provides examples). Pearson's correlation revealed significant positive relationships in both groups, with amplitude-velocity (control: *r* = 0.644, *p* < 0.0001; FASD: *r* = 0.613, *p* < 0.0001) and amplitude-duration (control: *r* = 0.471, *p* < 0.0001; FASD: *r* = 0.538, *p* < 0.0001) exhibiting the strongest relationships, followed by duration-velocity (control: *r* = 0.136, *p* < 0.0001; FASD: *r* = 0.103, *p* < 0.0001). The slopes of the main sequence relationships were calculated separately for each participant. The mean slopes of the amplitude-velocity relationship were different between groups with the FASD group displaying a significantly lower slope [*t*_(180)_ = 2.413, *p* = 0.0168; Figure 1B]. Thus, saccades produced by children with FASD tended to be slower than saccades produced by controls. The mean slopes of the amplitude-duration relationship were not significantly different (data not shown).

**Figure 2:**
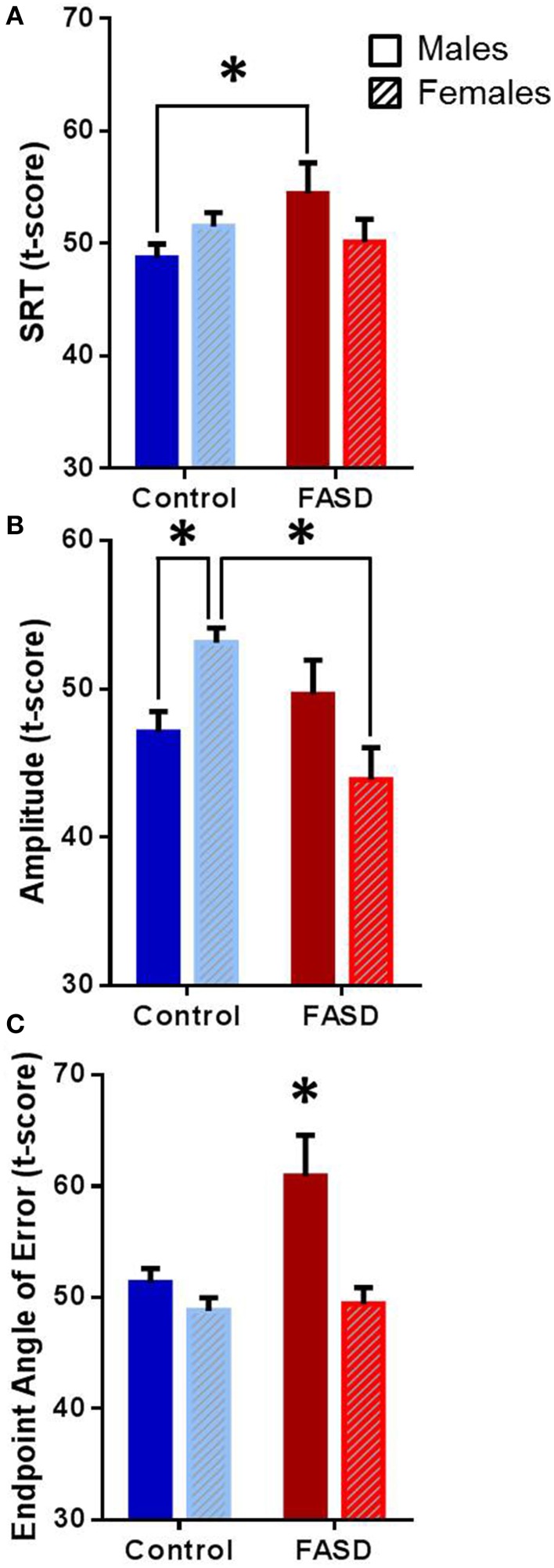
**Overall data**. Data are mean ± SEM for participants in the FASD group (*n* = 71) shown in red and the control group (*n* = 113) shown in blue. **(A)** Males with FASD had significantly slower saccadic reaction time (SRT) compared to control males. **(B)** Control females had significantly greater amplitude compared to control males and females with FASD. **(C)** Males with FASD had significantly greater endpoint angle of error compared to all other groups. ^*^p < 0.05 compared to group indicated or all other groups.

Several behavioral sexually dimorphic effects were found both within and between groups (Table 2). There was a significant interaction between group and sex [*F*_(1, 182)_ = 4.12, *p* = 0.044] for SRT (Figure 2A), and the *post-hoc* test revealed that males with FASD were slower than control males (*p* = 0.043). A main effect of group [*F*_(1, 180)_ = 4.016, *p* = 0.047] and an interaction between group and sex [*F*_(1, 180)_ = 12.54, *p* = 0.0005] were found for amplitude (Figure 2B). *Post-hoc* analysis revealed that females in the control group had greater saccade amplitude compared to control males (*p* = 0.020) and females with FASD (*p* = 0.0009). Main effects of group [*F*_(1, 182)_ = 12.11, *p* = 0.0006] and sex [*F*_(1, 182)_ = 6.39, *p* = 0.012], and an interaction [*F*_(1, 182)_ = 4.97, *p* = 0.027] were found for saccade endpoint angle of error (Figure 2C). The *post-hoc* test revealed that males with FASD had greater endpoint angle of error compared to control males (*p* = 0.0037) and both control females (*p* > 0.0001) and females with FASD (*p* = 0.0023).

**Table 2:** **Sex differences between FASD and controls**.

**Measure**	**Control (mean ± SEM)**		**FASD (mean ± SEM)**	
	**Males**	**Females**	**Males**	**Females**
SRT *t*-score	48.7 ± 1	51.5 ± 1	54.4 ± 3^†^	50.1 ± 2
Amplitude *t*-score	47.1 ± 1^*^	53.1 ± 1	49.6 ± 2	43.9 ± 2^*^
Endpoint *t*-score	51.3 ± 1	48.8 ± 1	60.9 ± 4^†^^*^	49.4 ± 1

* *Indicates significant difference from control females*,

† *indicates significant difference from control males*.

### Amplitude restricted data findings

**Figure 3:**
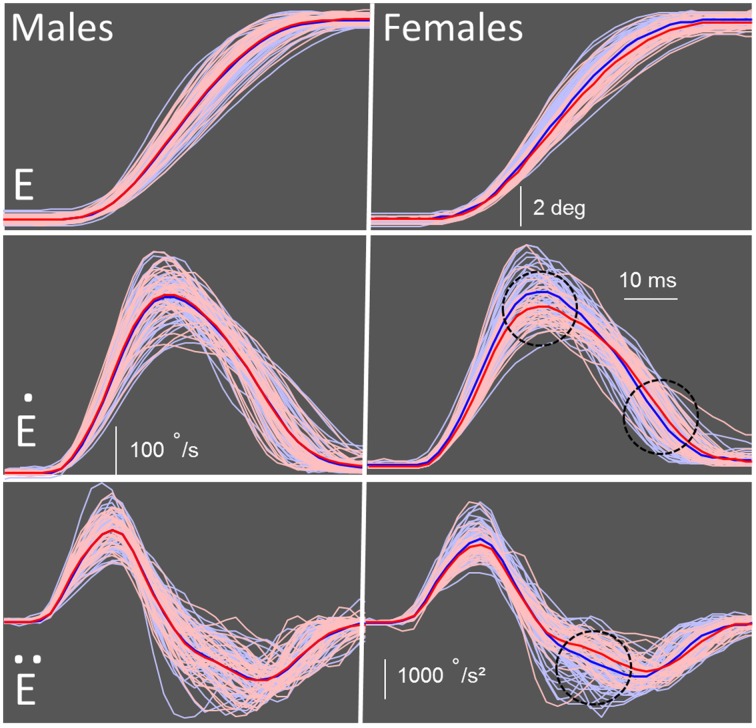
**Schematic of metric measures**. Individual data for each participant are shown in pink for the participants with FASD (*n* = 71) and light blue for the controls (*n* = 113). The mean of the FASD group is shown as a bold red line and control group as a bold blue line. Males (control vs. FASD) are on the left and females (control vs. FASD) are on the right. **(E)** Eye traces of correct saccades when participants look from a central fixation point to a peripheral target. **(Ė)** Velocity profiles of the control and FASD participants. Peak velocity was significantly slower in the females with FASD compared to control females. **(Ë)** Acceleration and deceleration profiles of all participants. Peak deceleration, but not acceleration, was significantly slower in the females with FASD compared to control females. ^*^Significant results (*p* < 0.05) are indicated by black dotted circle.

Due to the group difference we observed in amplitude and endpoint error, a proper metrics analysis could not be conducted. However, by matching saccade amplitude, the velocity and duration could be further examined. We restricted the amplitude range to 9 ± 1° and found that several group and sex differences emerged between the control and FASD groups (Table 3). There were no significant differences found for SRT. As expected, there was a significant interaction [*F*_(1, 180)_ = 5.19, *p* = 0.024] found for amplitude, but no *post-hoc* group or sex differences. There was a main effect of group [*F*_(1,180)_ = 5.67, *p =* 0.018] and an interaction between group and sex [*F*_(1, 180)_ = 5.05, *p* = 0.026] found for peak velocity (Figure 3). The *post-hoc* test revealed that the females in the FASD group had lower peak velocity when compared to control females (*p* = 0.0087). There was also a main effect of group [*F*_(1, 180)_ = 4.20, *p* = 0.042] found for duration with the FASD group displaying longer duration; however, there was no effect of sex or interaction between groups. Peak acceleration was not different between the groups, but a significant interaction between group and sex [*F*_(1, 180)_ = 4.55, *p* = 0.0344] was found for peak deceleration (Figure 3). The *post-hoc* test revealed that females in the FASD group had slower peak deceleration compared to control females (*p* = 0.030). There were no significant results found for mean acceleration, but there was a significant interaction between group and sex for mean deceleration [*F*_(1, 180)_ = 5.95, *p* = 0.0157]. The *post-hoc* test again revealed that females with FASD had a decreased mean deceleration compared to control females (*p* = 0.0072). There was a main effect of group [*F*_(1, 180)_ = 5.86, *p* = 0.023] and an interaction [*F*_(1, 180)_ = 4.01, *p* = 0.047] for skew index. The females with FASD had a greater skew index compared to both control males (*p* = 0.020) and females (*p* = 0.037). There were no significant results found for saccade endpoint error. Therefore, once amplitude was matched between the two groups several metric deficits were found between groups with the females with FASD displaying increased vulnerability.

**Table 3:** **Sex differences between FASD and control for amplitude restricted dataset**.

**Measure**	**Control (mean ± SEM)**		**FASD (mean ± SEM)**	
	**Males**	**Females**	**Males**	**Females**
Velocity (°/s)	327±4.9	339±4.9	327±6.4	313±5.2^*^
Amplitude (°)	9.0±0.04	9.2±0.04	9.1±0.06	9.0±0.06
Duration (ms)	49.7±0.8	48.8±0.5	51.2±1.2	50.6±0.9
Acceleration (°/s^2^)	23,958±551	25,527±555	23,990±702	23,659±646
Deceleration (°/s^2^)	−19,706±428	−20,823±430	−20,082±618	−19,032±528^*^
Slope 1	13.5 ± 0.4	14.2 ± 0.3	13.3 ± 0.4	13.4 ± 0.4
Slope 2	10.4 ± 0.4	11.1 ± 0.3	10.6 ± 0.3	9.7 ± 0.4^*^
Skew index	0.12 ± 0.01	0.12 ± 0.01	0.13 ± 0.01	0.17 ± 0.02^*^
SRT *t*-score	163.5 ± 5.4	167.7 ± 4.5	172.7 ± 6.8	162.6 ± 6.7
Endpoint (°)	2.5 ± 0.1	2.5 ± 0.1	2.8 ± 0.1	2.5 ± 0.1

* *Indicates significant difference from control females*.

## Discussion

The first objective of this study was to perform a more extensive examination of saccade metrics, in particular the main sequence, in children with FASD compared with typically developing controls. We found that children with FASD exhibited an alteration in the main sequence, specifically decreased slope of the velocity-amplitude relationship. This indicates that these two measures are linearly related in both groups with the FASD group displaying decreased velocities, leading to a decreased slope of the velocity-amplitude relationship. This pattern of deficits is a potential biomarker of impairment in either the brainstem circuitry or cerebellum of FASD participants.

The second objective was to test for sex differences where we found that males with FASD had slower SRT and greater saccade endpoint deviation, while the females with FASD had significantly decreased amplitude. When the performance of all amplitude restricted trials was examined, males with FASD did not show any differences from controls, but the females with FASD exhibited lower peak velocity, peak deceleration, and average deceleration. The females with FASD also had increased skewness of the velocity profile. Thus, the potential impairment in cerebellar and/or brainstem circuits may also have a sex component that should not be overlooked.

### Behavioral findings

Amplitude and velocity of saccadic eye movements exhibit a consistent relationship in which larger amplitude eye movements are accompanied by greater peak velocity in healthy controls (Boghen et al., 1974; Bahill et al., 1975; Leigh and Zee, 2006). This same main sequence relationship was found for both the FASD and control groups, but the slope of this relationship was reduced for the FASD group. Saccade duration is also linearly related to amplitude (Bahill et al., 1975; Baloh et al., 1975; Leigh and Zee, 2006). Again, this relationship held for both the FASD and control group but no differences in slopes were found between the two groups. This is not surprising because saccade duration was not significantly different between the two groups and a large range of amplitudes were not examined.

Only a few recent studies have reported sexually dimorphic differences in humans with FASD (e.g., Dodge et al., 2014; Fuglestad et al., 2014), however none have examined eye movement behaviors. Additionally, many animal studies have found both physiological and behavioral sex-dependent differences when examining prenatal alcohol exposure. For example, female rats prenatally exposed to alcohol have been found to have enhanced response to stressors (Halasz et al., 1993) and show deficits in their ability to use or respond to environmental cues. Increased impairment in female animals was also found for spatial working memory after prenatal alcohol exposure (Weinberg, 1992a,b). Finally, differences in recognition memory and social cues have also been found to be increased in female, but not male, rats exposed prenatally to alcohol (Kelly et al., 2009). These animal findings and the small number of human studies examining sex differences led us to investigate if any eye movement measures distinguished males and females prenatally exposed to alcohol.

Interestingly, both males and females with FASD did show sexually dimorphic results on prosaccade eye movement measures. The mechanism of how this occurs is unknown but animal studies have suggested that prenatal alcohol exposure may be altering the gonadal-adrenal interactions during fetal development (Weinberg et al., 2008; Carter et al., 2014). In our study, males with FASD had slower reaction times compared to control males. Slower SRTs have been previously found in a different population of children with FASD; however, interactions between sexes were not investigated (Green et al., 2007, 2009). Additionally, slower reaction time have also been found in children with FASD when completing other tasks (e.g., Jacobson et al., 1994; Kable and Coles, 2004; Burden et al., 2005). Increases in SRT can be caused by damage to many different structures in the brain and can also indicate diffuse cortical damage in regions such as the occipital, frontal, and parietal lobes (Leigh and Zee, 2006). Therefore, it appears that males with FASD may have less specific but more widespread and diffuse damage due to prenatal alcohol exposure, leading to increased SRT.

Sexually dimorphic results also emerged when saccadic trajectory and accuracy were examined in the unrestricted data. Saccades have very short durations, and because of this there is insufficient time for visual feedback to correct ongoing movements, and therefore inaccuracies can be caused by deficits in internal monitoring (Leigh and Kennard, 2004). Increased variability in trajectory can also lead to saccade inaccuracies (Smit and Van Gisbergen, 1990; Van der Stigchel et al., 2006). Finally, saccade inaccuracies can be caused by overshooting (hypermetric) or undershooting (hypometric) the target or object of interest. In this study, females with FASD produced smaller initial amplitude saccades compared to control females, and males with FASD displayed greater endpoint inaccuracy compared to both control males and females. This indicates that whereas both sexes in the FASD group were less accurate, the underlying cause of the inaccuracy differed, suggesting sex differences in brain injury due to prenatal alcohol exposure.

After restricting the data by matching amplitudes, the velocity, duration and saccade waveform could be properly examined (Figure 3). The analysis revealed a group but not sex difference in duration. There was both a group and sex difference in peak velocity and peak deceleration with females with FASD displaying a decrease in both. In healthy controls, the skewness of the velocity waveform has been consistently found to be asymmetrical during a horizontal saccade, with a skew to the left (Baloh et al., 1975; Van Opstal and Van Gisbergen, 1987). In the current study, a more positive skew was found for the females with FASD compared to both control males and females. This is caused by the decreased mean deceleration of the velocity profile.

### Neural mechanisms associated with saccade impairments

The saccade inaccuracies observed in the initial saccades of the unrestricted dataset implicate the cerebellum (Keller et al., 1983; Crawford and Guitton, 1997; Leigh and Zee, 2006; Collins et al., 2008). The nucleus reticularis tegmenti pontis encodes the size and direction of saccades in three dimensional eye displacement vectors (Van et al., 1996). It projects to the dorsal vermis and caudal fastigial nucleus of the cerebellum. The dorsal vermis is involved in modulating on-line amplitude and trajectory during a saccade (Keller et al., 1983). The fastigial nucleus works with the dorsal vermis to control saccade accuracy by monitoring motor commands via internal feedback of the desired and ongoing motor command (efference copy) and corrections for anticipated errors are produced by subtle yet rapid modifications of saccade duration (Robinson and Fuchs, 2001). These connected structures play a critical role in saccade metrics and accuracy and we propose that they appear to be impacted by prenatal alcohol exposure.

After restricting the dataset and matching saccade amplitude, the neural correlates of both velocity and duration could be examined in greater detail. The decreased peak velocity observed in females with FASD may indicate damage to the brainstem itself or its connections because the pons is critical for the generation of saccades. Specifically, excitatory burst neurons in the paramedian pontine reticular formation are essential for driving the initial acceleration to generate a horizontal saccade (Scudder et al., 1996; Leigh and Zee, 2006). Therefore, the decreased peak velocity found in females with prenatal alcohol exposure may be due to damage to the paramedian pontine reticular formation.

In addition to the decreased peak velocity observed in the females with FASD, it also appears that when the saccade waveform was analyzed in the restricted dataset, females with FASD had deficits specific to the latter half of the saccade. Inactivation of the caudal fastigial nucleus using muscimol is known to decrease deceleration with little to no effect on acceleration (Robinson et al., 1993; Goffart et al., 2004). The caudal fastigial nucleus projects to the burst neurons in the pons and this projection may be impaired in females with FASD (Goffart et al., 2004; Buzunov et al., 2013). The deficit in the peak and mean deceleration in the females with FASD is indicative of cerebellar damage in the caudal fastigial nucleus or its projections to the brainstem.

## Conclusions

The findings reported here indicate that children with FASD have deficits in multiple measures of saccade performance including accuracy, main sequence, and SRT. Additionally, we conclude, for the first time, that prenatal alcohol exposure has a sexually dimorphic impact on eye movement control with females exhibiting greater metric vulnerability and males exhibiting greater variability in SRTs and online error correction. These findings implicate impairment in cerebellar and/or brain stem circuits. The next steps will be to combine the current measures with MRI studies to better characterize the neural correlates of the sexually dimorphic results. Additionally, males and females with FASD appear to have distinct patterns of saccade measure deficits that may be used to screen for those prenatally exposed to alcohol using a simple 5 min saccade task. Earlier diagnosis has been found to be crucial for children with FASD because it leads to better outcomes later in life due to the recognition and treatment for neurological, behavioral, and mental health issues faced by these children.

### Conflict of interest statement

The authors declare that the research was conducted in the absence of any commercial or financial relationships that could be construed as a potential conflict of interest.
